# Supplementary figures and images for: Functional Connectivity between the Cerebellum and Somatosensory Areas Implements the Attenuation of Self-Generated Touch
Source: J Neurosci. 2020 Jan 22;40(4):894–906. doi: 10.1523/JNEUROSCI.1732-19.2019 (PMC6975290; doi:10.1523/JNEUROSCI.1732-19.2019)

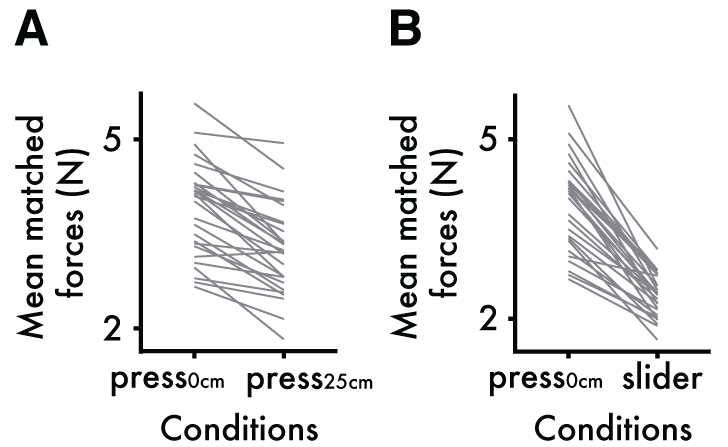

Supplement: Figure 1-1 [file sup_ns-JN-RM-1732-19-s18.tif]

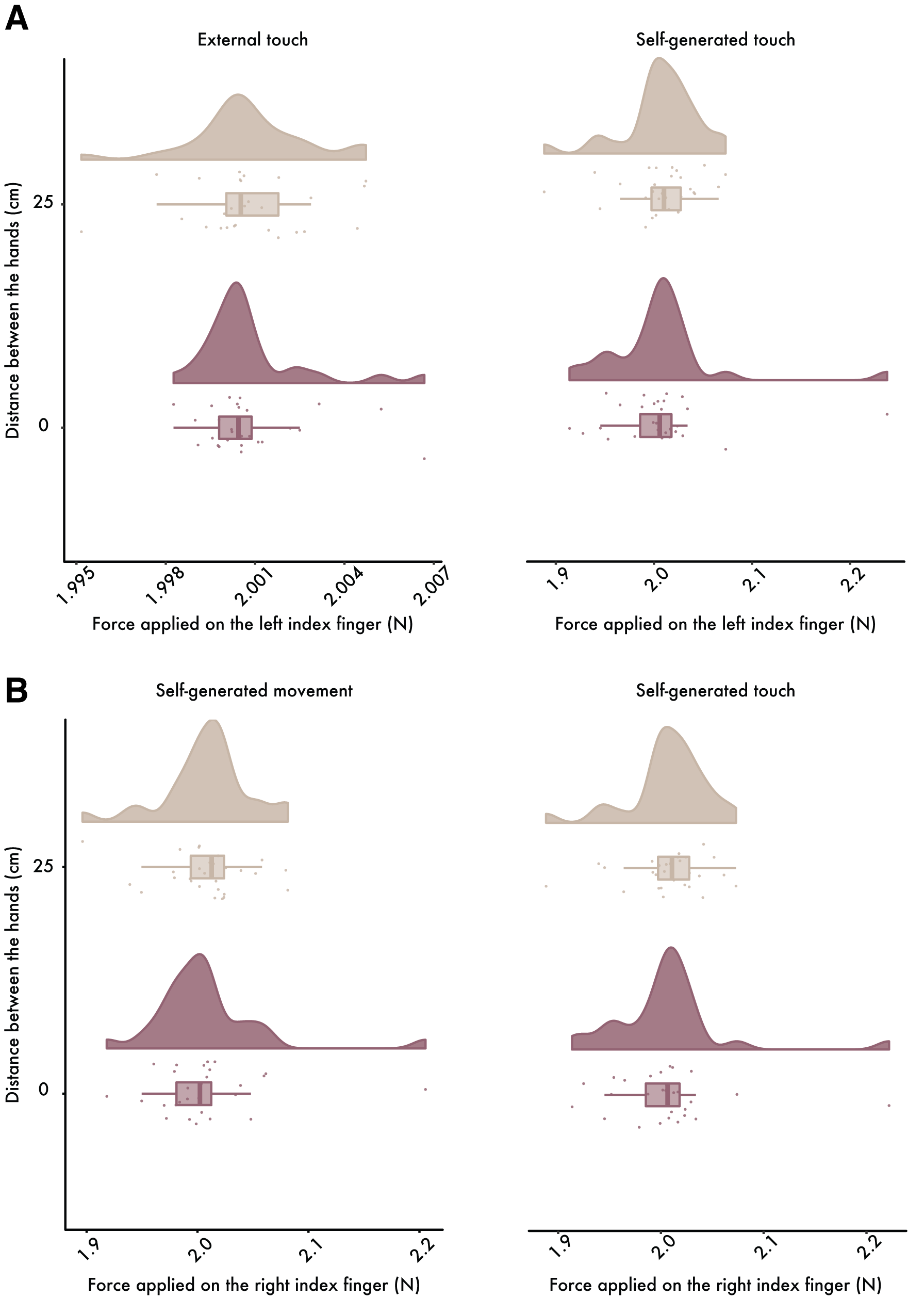

Supplement: Figure 2-1 [file sup_ns-JN-RM-1732-19-s19.tif]

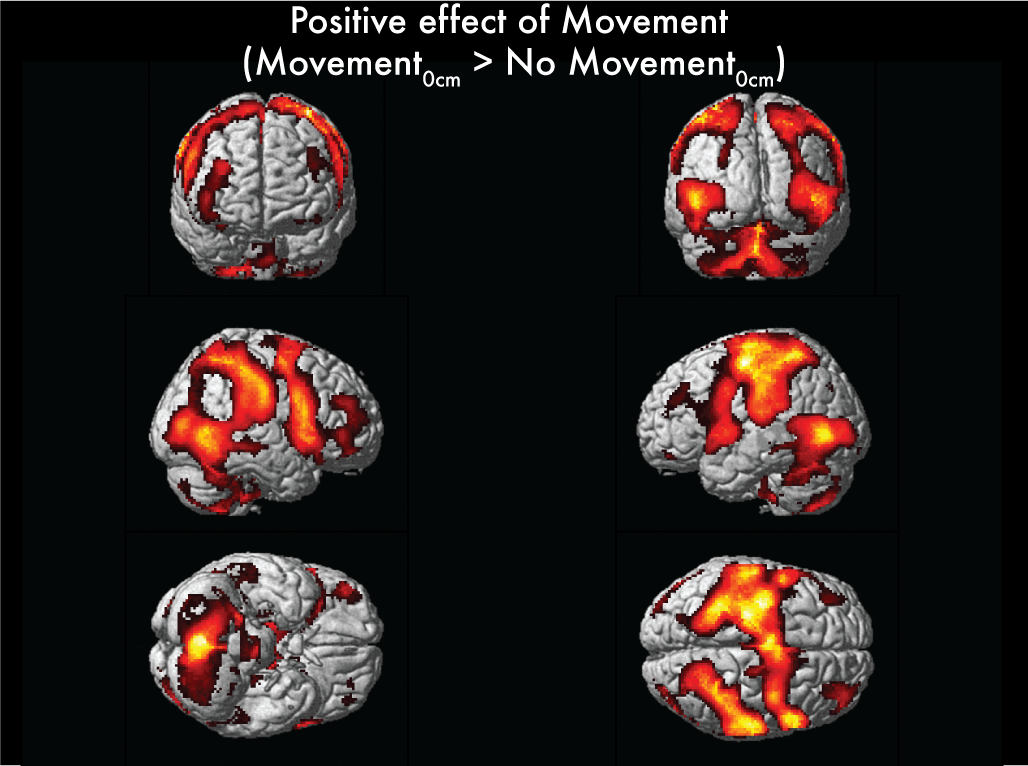

Supplement: Figure 3-1 [file sup_ns-JN-RM-1732-19-s20.tif]

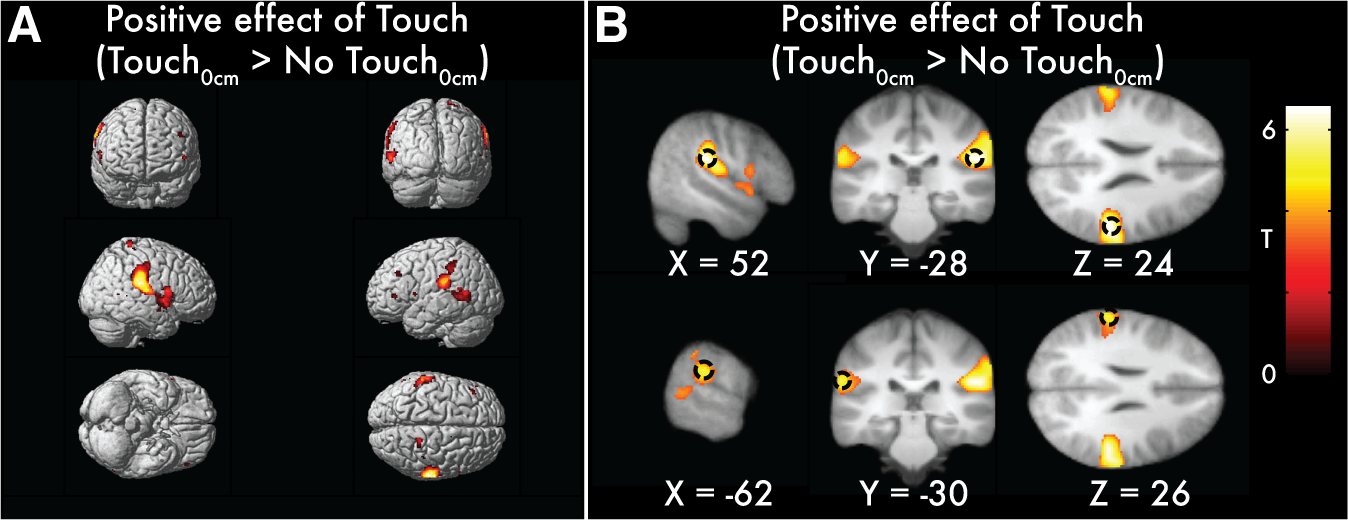

Supplement: Figure 3-2 [file sup_ns-JN-RM-1732-19-s21.tif]

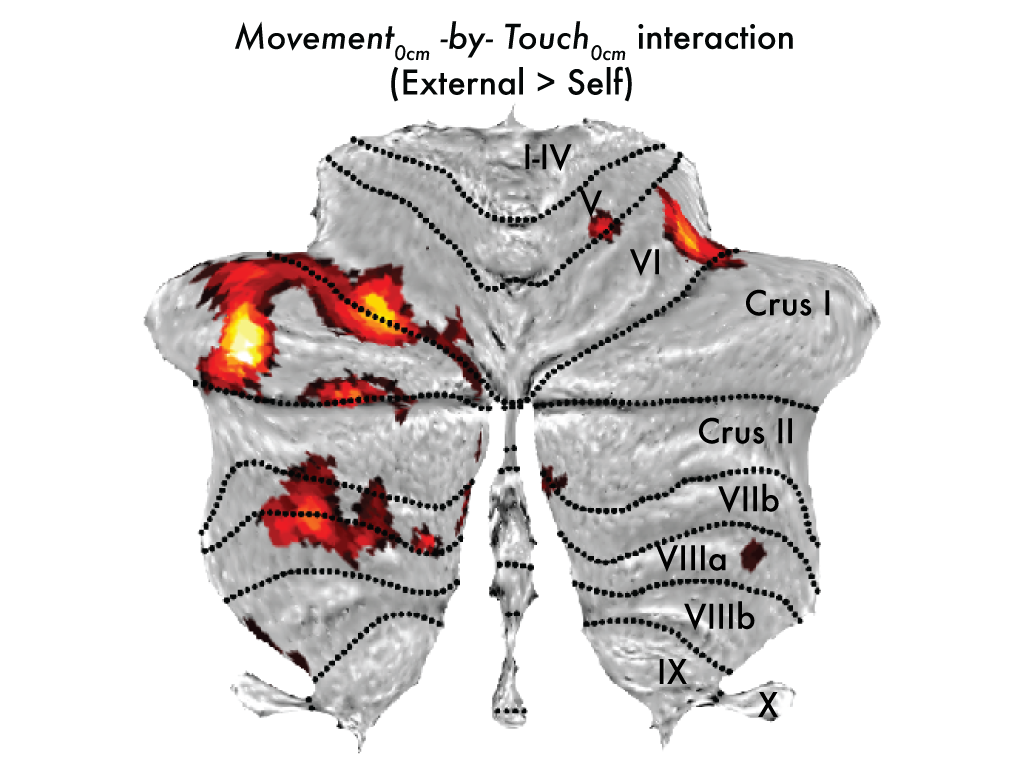

Supplement: Figure 3-3 [file sup_ns-JN-RM-1732-19-s22.tif]

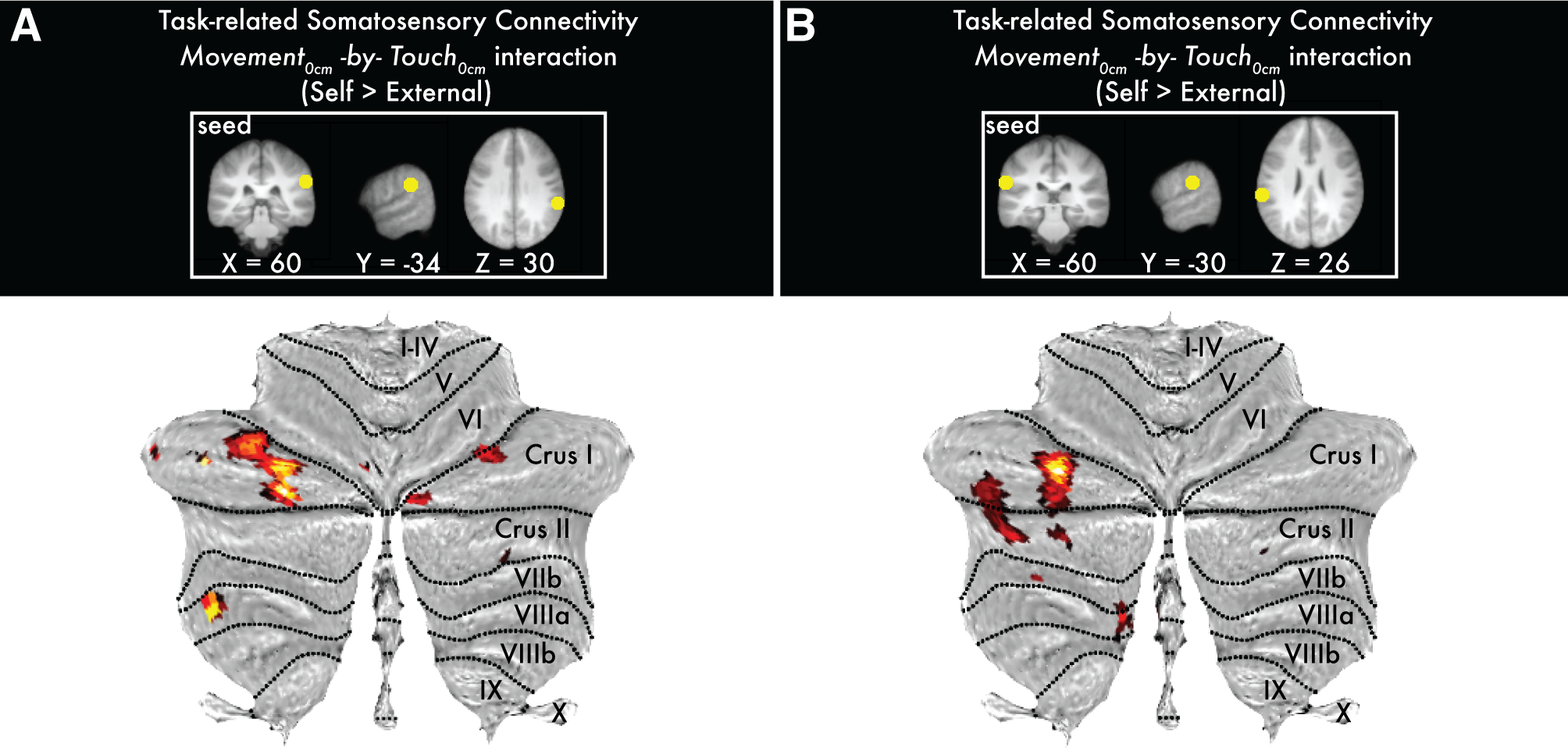

Supplement: Figure 4-1 [file sup_ns-JN-RM-1732-19-s23.tif]

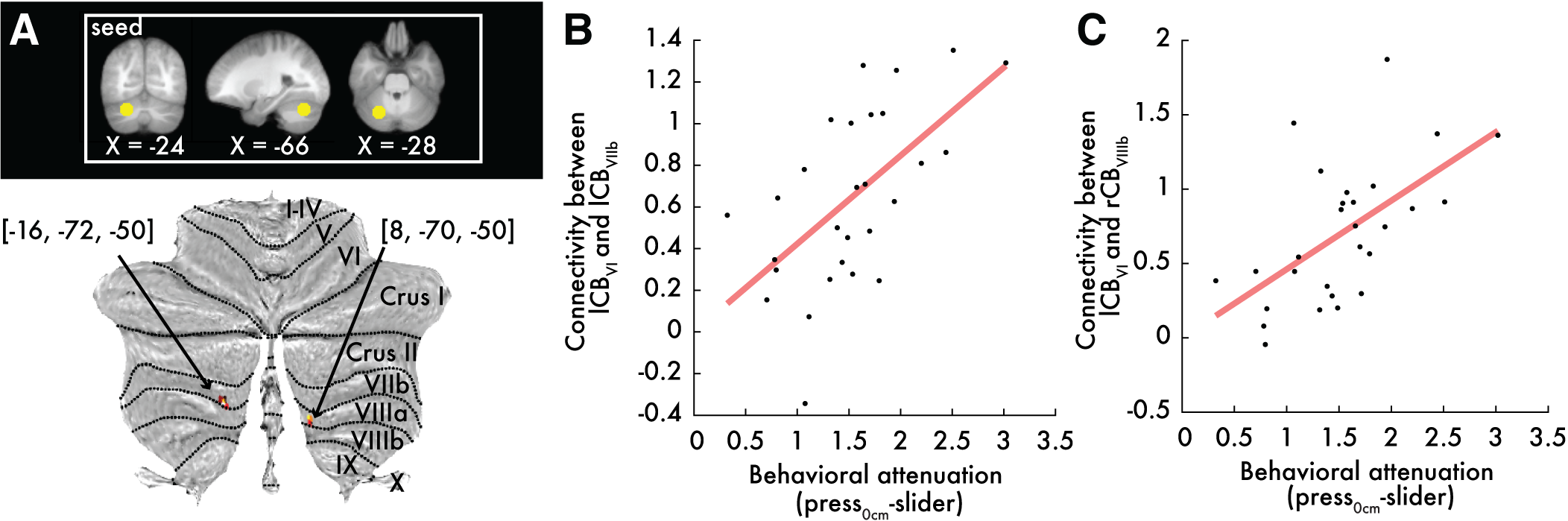

Supplement: Figure 4-2 [file sup_ns-JN-RM-1732-19-s24.tif]

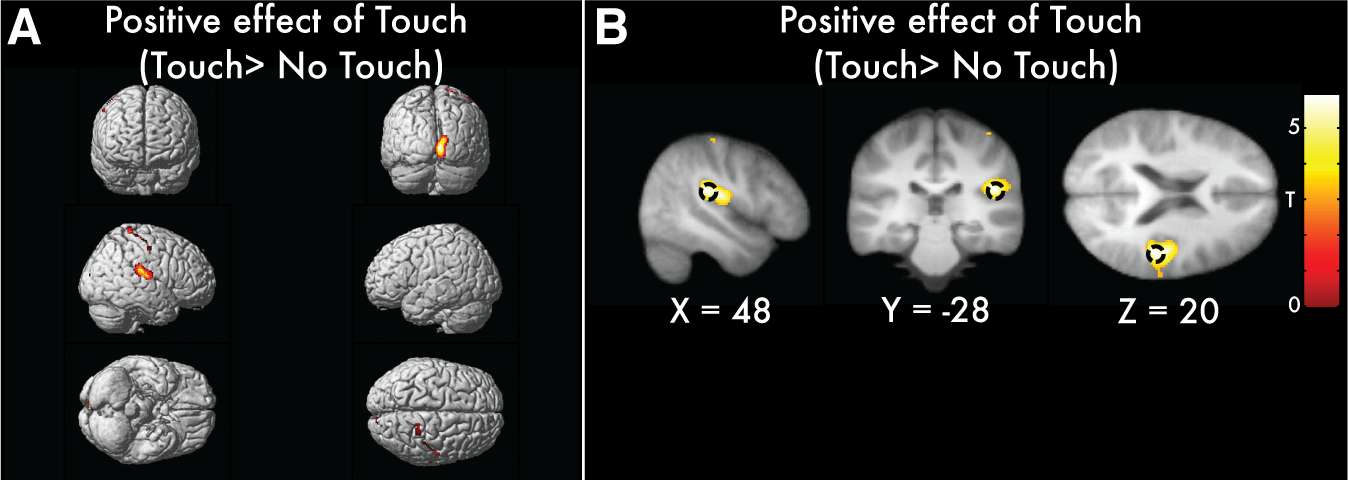

Supplement: Figure 5-1 [file sup_ns-JN-RM-1732-19-s25.tif]

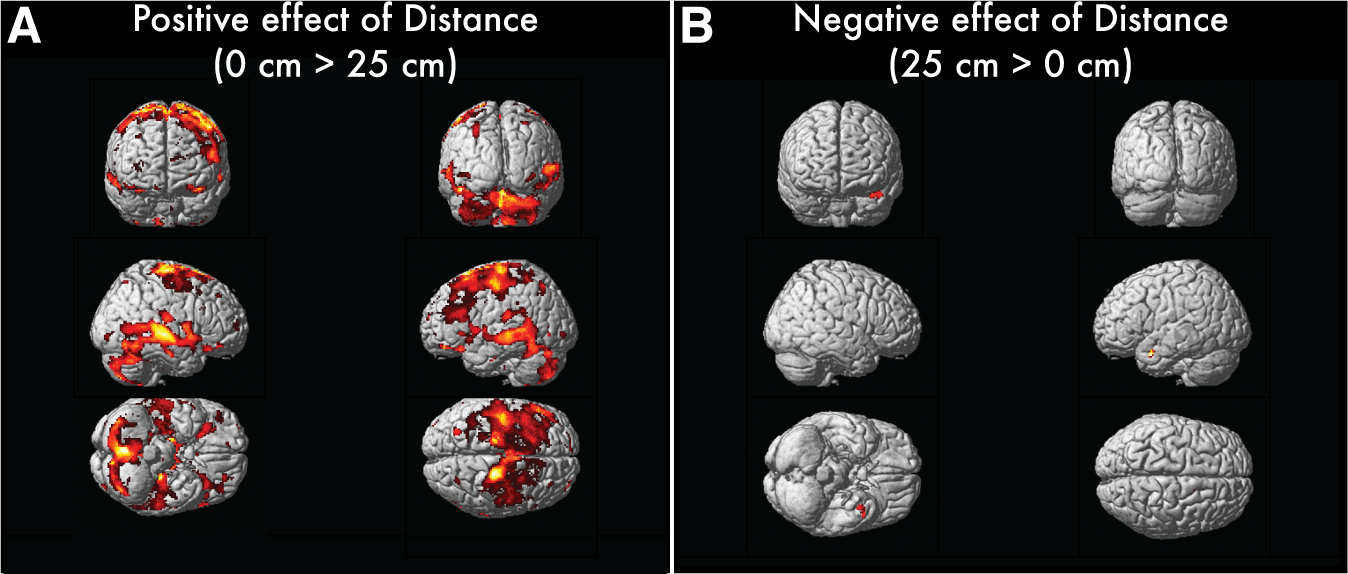

Supplement: Figure 5-3 [file sup_ns-JN-RM-1732-19-s26.tif]

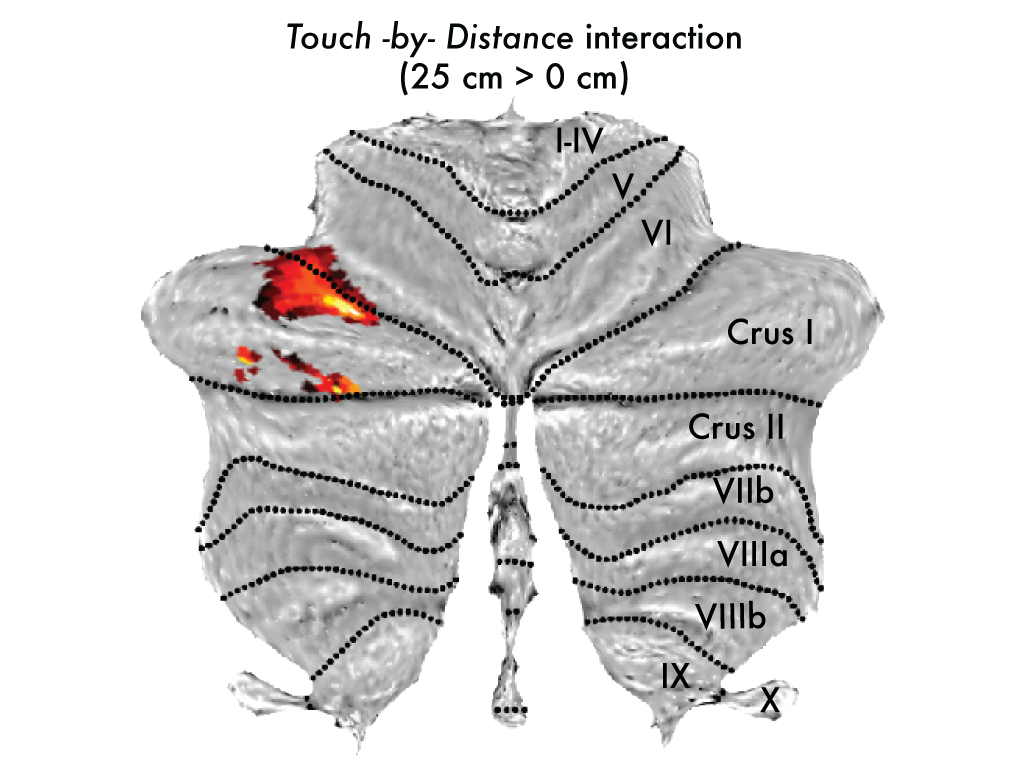

Supplement: Figure 5-7 [file sup_ns-JN-RM-1732-19-s27.tif]

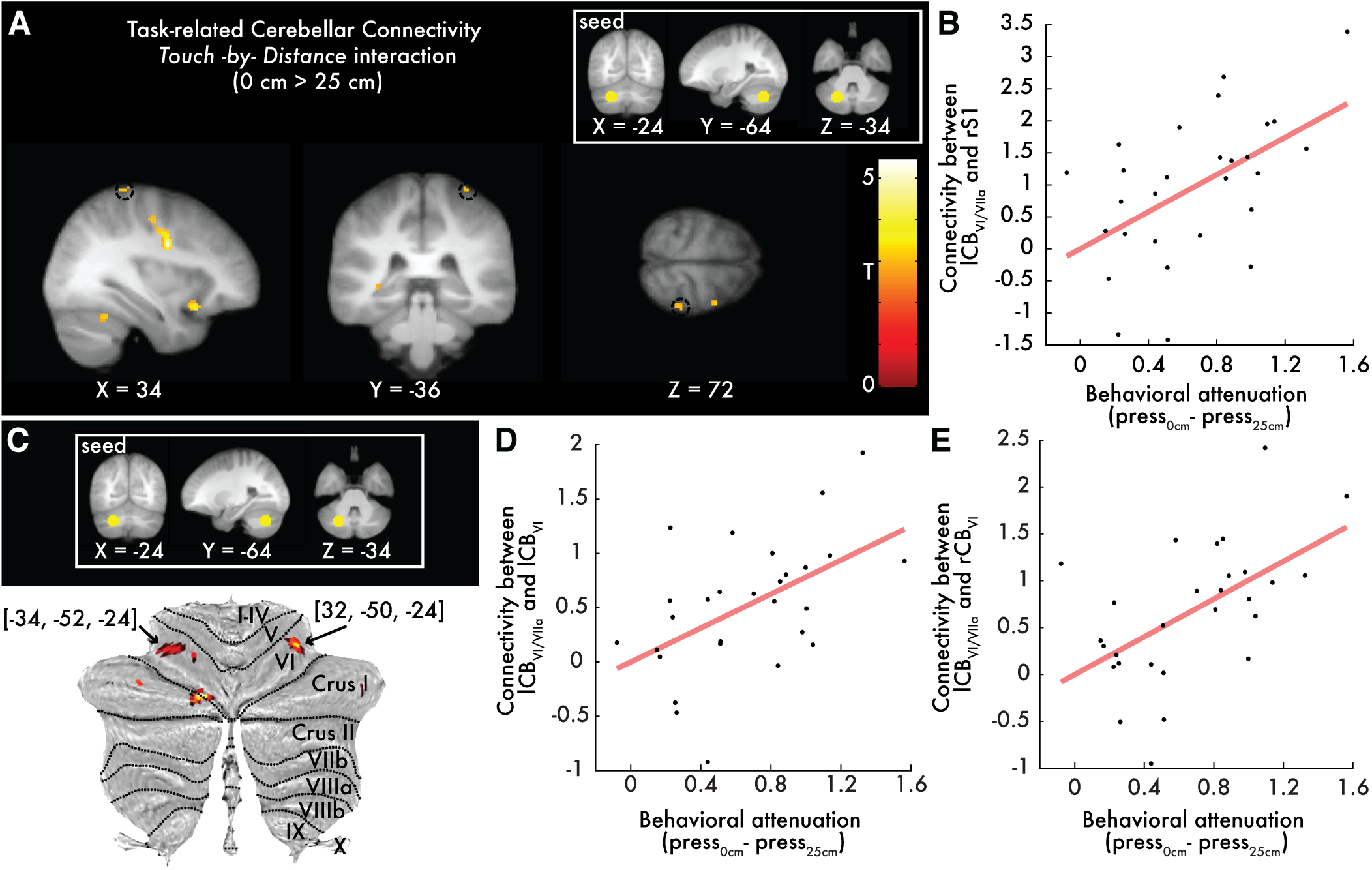

Supplement: Figure 5-9 [file sup_ns-JN-RM-1732-19-s28.tif]

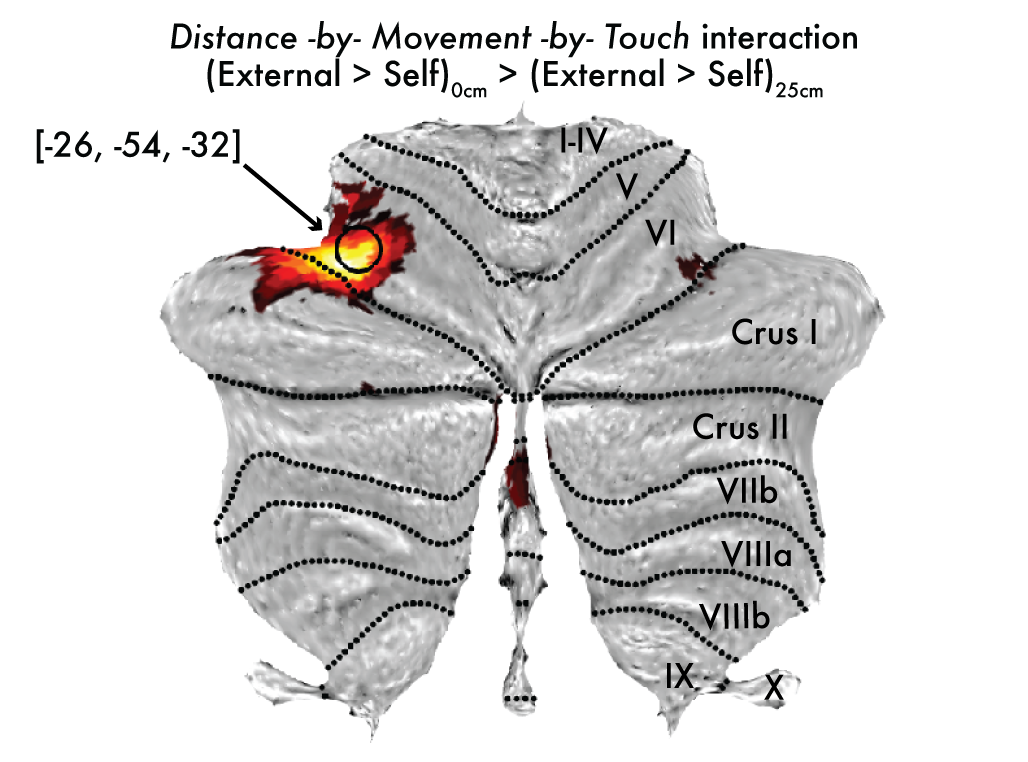

Supplement: Figure 5-12 [file sup_ns-JN-RM-1732-19-s29.tif]
